# From the bench to the clinic: basophils and type 2 epithelial cytokines of thymic stromal lymphopoietin and IL‐33

**DOI:** 10.1002/cti2.70020

**Published:** 2024-12-09

**Authors:** Kazushige Obata‐Ninomiya, Tharmalingam Jayaraman, Steven F Ziegler

**Affiliations:** ^1^ Center of Fundamental Immunology Benaroya Research Institute Seattle WA USA; ^2^ Department of Biomedical Engineering University of Houston Houston TX USA; ^3^ Department of Immunology University of Washington School of Medicine Seattle WA USA

**Keywords:** basophil, IL‐33, thymic stromal lymphopoietin, type 2 immunity

## Abstract

Type 2 epithelial cytokines, including thymic stromal lymphopoietin and IL‐33, play central roles in modulation of type 2 immune cells, such as basophils. Basophils are a small subset of granulocytes within the leukocyte population that predominantly exist in the blood. They have non‐redundant roles in allergic inflammation in peripheral tissues such as the lung, skin and gut, where they increase and accumulate at inflammatory lesions and exclusively produce large amounts of IL‐4, a type 2 cytokine. These inflammatory reactions are known to be, to some extent, phenocopies of infectious diseases of ticks and helminths. Recently, biologics related to both type 2 epithelial cytokines and basophils have been approved by the US Food and Drug Administration for treatment of allergic diseases. We summarised the roles of Type 2 epithelial cytokines and basophils in basic science to translational medicine, including recent findings.

## Graphical abstract

It has been well described that basophils and type 2 epithelial cytokines play important roles in allergic diseases. Based on these findings, significant progress has been made in translational research into these diseases. In this review, we summarise fundamental and novel findings that connect basic and translational research.

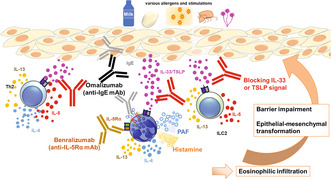

## Introduction

**Figure 1:**
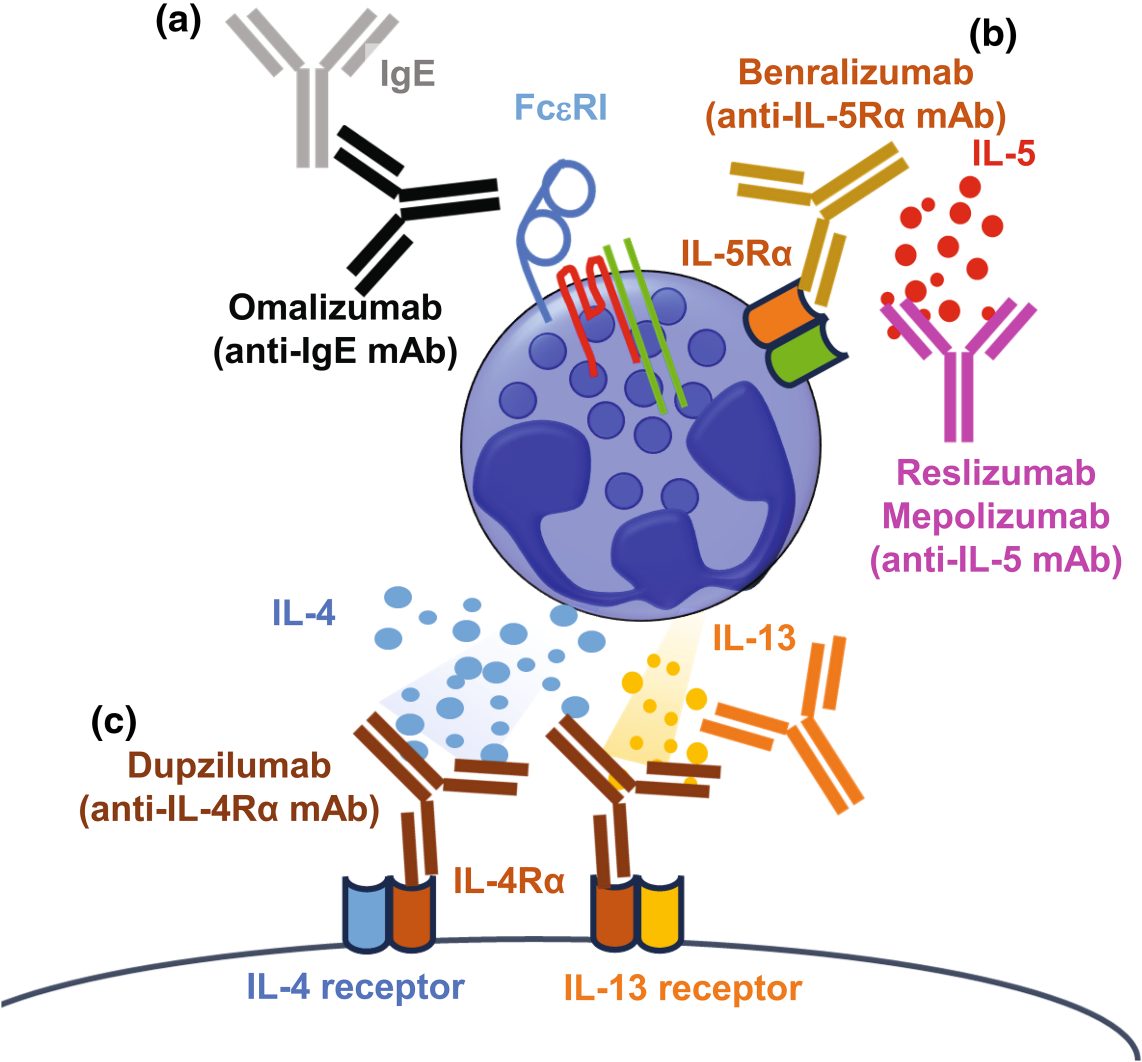
Biologics related to basophils. **(a)** Omalizumab binds to free IgE to inhibit binding to FcεRI on basophils. **(b)** Benzralizumab binds to IL‐5Rα and Mepolizumab binds to free IL‐5 to inhibit interaction between IL‐5 receptor and IL‐5. **(c)** Dupilumab binds to IL‐4Rα to block downstream effects of both IL‐4 and IL‐13. Taralokinumab binds to free IL‐13 to inhibit the interaction with its receptor.

Basophils are one of the rarest granulocytes in peripheral blood leukocytes. They produce chemical mediators such as histamine and secrete prostaglandins and proinflammatory cytokines, which lead to inflammatory reactions, contribute to allergic inflammation including atopic dermatitis and pulmonary diseases and also protect against helminth infections.^1^, ^2^, ^3^, ^4^, ^5^, ^6^, ^7^, ^8^ Biologics are medicines that have been isolated from natural sources, although they may be produced by artificial methods. Basophil‐related biologics such as IgE (Omalizumab), IL‐4 receptor α chain (Dupilumab), IL‐5 (Reslizumab and Mepolizumab), IL‐5 receptor α chain (Benralizumab) and IL‐13 (Tralokinumab) have been approved by the US Food and Drug Administration (FDA) for the treatment of allergic diseases (Table 1, Figure 1).

**Table 1:** US Food and Drug Administration (FDA) approved biologics related to basophils

Characteristic	Therapeutic target	Mechanism of action	Indications	Prescribing information
Omalizumab (XOLAIR^®^)	IgE	Binds to free IgE, inhibits binding to FcεRI on mast cells, basophils, and plasmacytoid dendritic cells; FcεRII on dendritic cells and eosinophils	Moderate–severe asthma and allergic asthma; chronic idiopathic urticaria; nasal polyps; food allergies	https://www.gene.com/download/pdf/xolair_prescribing.pdf
Dupilumab (DUPIXENT^®^)	IL‐4Ra	Binds to IL‐4Ra, blocking the downstream effects of both IL‐4 and IL‐13	Moderate–severe asthma with an eosinophilic phenotype; moderate‐severe atopic dermatitis; chronic RSwNP; eosinophilic esophagitis	https://www.accessdata.fda.gov/drugsatfda_docs/label/2017/761055lbl.pdf
Tralokinumab (ADBRYTM)	IL‐13	Binds to free/circulating IL‐13, thereby inhibiting interaction with its receptor	Moderate–severe atopic dermatitis in adults	https://www.accessdata.fda.gov/drugsatfda_docs/nda/2022/761180Orig1s000lbl.pdf
Mepolizumab (NUCALA)	IL‐5	Binds to free/circulating IL‐5, thereby inhibiting interaction with its receptor	Severe asthma with an eosinophilic phenotype; EGPA	https://www.accessdata.fda.gov/drugsatfda_docs/label/2019/761122s000lbl.pdf
Reslizumab (Cinqair)	IL‐5	Binds to free/circulating IL‐5, thereby inhibiting interaction with its receptor	Severe asthma with an eosinophilic phenotype	https://www.accessdata.fda.gov/drugsatfda_docs/label/2016/761033lbl.pdf
Benralizumab (FASENRA^®^)	IL‐5Ra	Binds to the IL‐5Ra chain, resulting in rapid depletion of eosinophils via antibody‐dependent cellular cytotoxicity	Severe asthma with an eosinophilic phenotype	https://www.accessdata.fda.gov/drugsatfda_docs/label/2017/761070s000lbl.pdf
Tezepelumab (TEZSPIRE^®^)	TSLP	Binds to TSLP, thereby inhibiting interaction with its receptor	Add‐on maintenance treatment in severe asthma	https://www.accessdata.fda.gov/drugsatfda_docs/label/2021/761224s000lbl.pdf

Type 2 epithelial cytokines are produced mainly from epithelial cells, induce type 2 immune responses and are associated with allergic disease and helminth infection.^9^ It has been well established that basophils are involved in thymic stromal lymphopoietin (TSLP) and IL‐33‐mediated response to allergens and helminths.^5^, ^10^, ^11^ The role of IL‐25 has also been discussed in relation to the functions of basophils.^12^, ^13^ Specifically, prior studies have shown that basophils are a source of IL‐25, express IL‐4 and IL‐13 and upregulate surface markers in response to IL‐25 through their IL‐25 receptors; however, another report showed that basophils do not respond to IL‐25.^12^, ^14^, ^15^ Despite these findings, we have not found any clinical trials targeting IL‐25 and its receptor in basophil‐related diseases. In this review, we focus on both basic and clinical research investigating basophils and type 2 epithelial cytokines of TSLP and IL‐33.

Research indicates that basophils display some activities in homeostasis and have immune suppressive functions.^16^, ^17^ Specifically, mouse basophils produce Amphiregulin, which activates regulatory T cells (Tregs) in mouse models of UVB‐induced suppression of contact hypersensitivity, IL‐4 and other factors that support the resolution of inflammation in atopic skin inflammation, improve myocardial infarction and prime group 2 innate lymphoid cells (ILC2s) in response to neuron‐derived signals for homeostasis of tissue integrity.^18^, ^19^, ^20^ Conversely, human Tregs activate basophils to produce pro‐inflammatory cytokines and upregulate expression of basophil activation markers such as CD69 and CD203c *in vitro*.^19^, ^21^ Additionally, the role of basophils has been discussed in the progression of several cancers, but the relationship with TSLP and IL‐33 remains to be discovered.^22^, ^23^, ^24^ Understanding novel functions of basophils may help to better understand the outcomes from biologics related to basophils in clinical trials.

## Thymic stromal lymphopoietin

TSLP belongs to the IL‐2 cytokine family, has four α‐helix bundle structures,^25^ and is expressed by epithelial cells at the barrier surfaces of organs including the lungs, skin and gastrointestinal tract in both homeostatic and inflammatory conditions.^26^, ^27^, ^28^, ^29^ Haematopoietic cells, including dendritic cells, basophils and mast cells, are also sources of TSLP.^30^, ^31^, ^32^ Mechanical injury, infection, inflammatory cytokines and proteases such as trypsin and papain can initiate release of TSLP from epithelial cells.^26^, ^33^, ^34^ Two different isoforms of TSLP are known in humans. The long isoform is upregulated in human bronchial epithelial cells and keratinocytes by stimulation with toll‐like receptor ligands, whereas the short isoform is mostly decreased or unaltered by those stimulations. It is unknown whether mice have multiple isoforms; however, administration of the synthetic short form TSLP reduced house dust mite‐induced inflammatory response in mice and prevented signalling of long form TSLP.^35^

The receptor for TSLP is composed of a TSLP Receptor (TSLPR) subunit and IL‐7 receptor α chain.^25^ TSLP signalling has both homeostatic and inflammatory functions. TSLP has anti‐apoptotic effects to induce expression of Bcl‐xL in primary human bronchial epithelial cells and Bcl‐2 in both human and mouse breast cancer cells.^36^, ^37^ Growth of human cervical cancer cells is associated with TSLP autocrine.^38^ Additionally, TSLP also contributes to anti‐apoptotic effects on intestinal epithelial cells in both humans and mice through induction of an endogenous inhibitor, secretory leukocyte peptidase inhibitor (SLPI), for neutrophil elastase that contributes to inflammatory bowel diseases.^39^, ^40^, ^41^, ^42^ TSLP has also been implicated in regulating Tregs in the skin, thymus and gut, leading to anti‐inflammatory effects in both humans and mice.^43^, ^44^, ^45^ Dendritic cell (DC)‐derived TSLP is important for mouse gut homeostasis.^46^, ^47^ TSLP signalling on DCs promotes Tregs in the gut to prevent bacteria‐mediated inflammation and TSLP signalling via interaction between DCs and T cells promotes Treg development, resulting in protection against colitis in a mouse model.^45^, ^47^ Intriguingly, TSLP‐responding Tregs are associated with progression of colorectal cancer in both humans and mice.^48^

TSLP also initiates inflammation via type 2 immune responses in haematopoietic cells such as eosinophilia.^49^ TSLP signalling induces activation of DCs to differentiate T helper 2 (Th2) cells through upregulation of OX40L.^50^ TSLP acts on T cells and B cells to modulate T‐cell differentiation and antibody production, respectively.^51^, ^52^

In some models, ILC2s are critical for TSLP‐associated allergic inflammation, especially steroid‐resistant allergic airway inflammation.^53^, ^54^, ^55^, ^56^, ^57^, ^58^ Patients with atopic allergic diseases such as AD, asthma, allergic rhinoconjunctivitis and eosinophilic esophagitis (EoE) are known to have dysregulated expression of TSLP linked to genetic variants of TSLP.^59^, ^60^ Overexpression of TSLP has been reported in AD, Netherton syndrome, asthma, COPD and EoE.^27^, ^61^ Some sensory neurons expressing TSLPR can drive the itch reaction in allergic diseases such as atopic dermatitis (AD) in response to TSLP.^62^, ^63^ Blockage of TSLP signalling has been tested in clinical trials in several diseases such as asthma, atopic dermatitis, cat allergy and EoE (Table 2). Tezepelumab (anti‐TSLP antibody) was approved for severe, uncontrolled asthma by the FDA in 2022.^64^ Tezepelumab has been tested in a Phase 3 trial (NCT05583227) for efficacy and safety in patients with EoE. In another trial, subcutaneous Tezepelumab did not reach statistically significant improvements in patients with moderate‐to‐severe atopic dermatitis.^65^

**Table 2:** Clinical trials of biologics targeting TSLP (from ClinicalTrials.gov, accessed 15 November 2024)

Study title	Study status	Conditions	Biologics	Character of biologics	Phases	Identifier
A study to evaluate the safety and efficacy of GR2002 injection in patients with atopic dermatitis	Not yet recruiting	Atopic dermatitis	GR2002 injection	Anti‐TSLP bispecific antibody	Phase 1	NCT06175143
First‐in‐human study of SAR443765 in healthy participants and in asthmatic participants	Completed	Asthma	SAR443765	Bifunctional nanobody blocking TSLP and IL‐13	Phase 1	NCT05366764
Single ascending doses study of anti‐interleukin‐7 receptor α monoclonal antibody (GSK2618960) in healthy volunteers	Completed	Autoimmune diseases	GSK2618960	Anti‐IL‐7 receptor a mAb	Phase 1	NCT02293161
Anti‐TSLP (AMG 157) plus antigen‐specific immunotherapy for induction of tolerance in individuals with cat allergy	Completed	Cat allergy Cat hypersensitivity	AMG 157	Anti‐TSLP mAb	Phase 1 Phase 2	NCT02237196
A extension clinical study of TQC2731 injection in the treatment of chronic sinusitis with nasal polyps	Recruiting	Chronic rhinosinusitis with nasal polyps	TQC2731	Anti‐TSLP mAb	Phase 2	NCT06451640
Tezepelumab in allergic rhinitis and asthma study (TEZARS)	Recruiting	Asthma with allergic rhinitis	Tezepelumab	Anti‐TSLP mAb	Phase 2	NCT06189742
A clinical study of TQC2731 injection in the treatment of chronic rhinosinusitis with nasal polyps	Recruiting	Chronic sinusitis nasal polyps	TQC2731 injection	Anti‐TSLP mAb	Phase 2	NCT06036927
Tezepelumab and methacholine airway hyperresponsiveness in participants with mild allergic asthma	Not yet recruiting	Asthma, allergic	Tezepelumab	Anti‐TSLP mAb	Phase 2	NCT05740748
Effects of blocking TSLP on airway inflammation and the epithelial immune‐response to exacerbation triggers in patients with COPD	Recruiting	COPD COPD exacerbation COPD bronchitis airway disease|immune system disorder	Tezepelumab	Anti‐TSLP mAb	Phase 2	NCT05507242
Evaluate the efficacy and safety of TQC2731 injection in patients with severe asthma	Unknown	Severe asthma	TQC2731	Anti‐TSLP mAb	Phase 2	NCT05472324
Effects of anti‐TSLP in patients with asthma	Completed	Asthma	MEDI9929	Anti‐TSLP mAb	Phase 2	NCT02698501
Tezepelumab on airway structure and function in patients with uncontrolled moderate‐to‐severe asthma	Recruiting	Asthma	Tezepelumab	Anti‐TSLP mAb	Phase 3	NCT05280418

## TSLP and basophils

**Figure 2:**
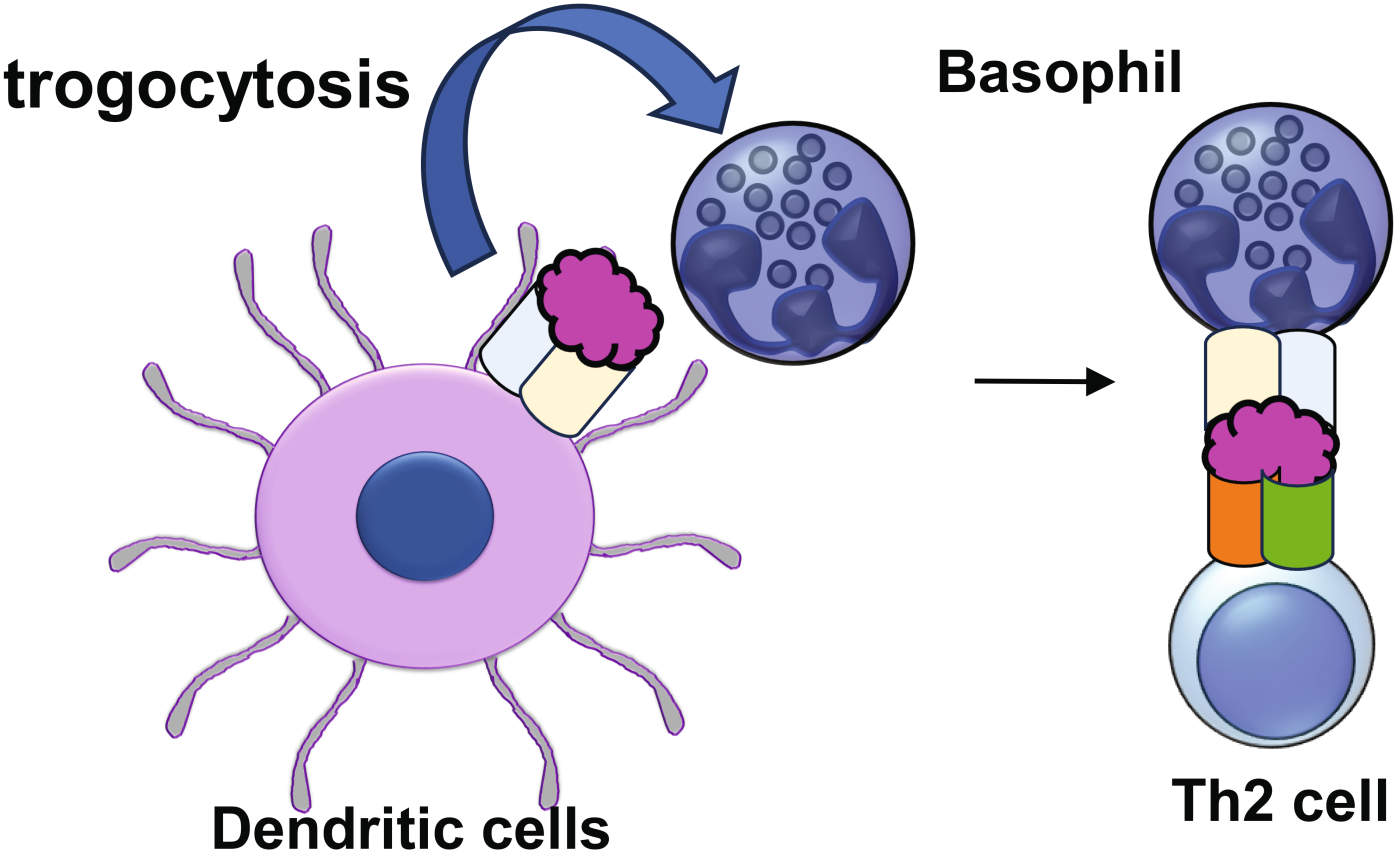
Basophils are acquired to express MHC class II complexes by trogocytosis from dendritic cells after treatment of mice with MC903, leading to induction of Th2 cells.

The role of basophils has been investigated in TSLP‐dependent inflammation in mice, using topical vitamin D3 analogue (MC903)‐induced skin inflammation, which is akin to atopic dermatitis in humans. In this model, basophils lacking expression of IL‐4, *Il4* 3′ untranslated region (Il4 3′UTR) mice, exhibit impairment of skin inflammation including swelling, reduction of levels of antigen‐specific IgE in serum and production of type 2 cytokines in draining lymph nodes.^66^ Human basophils in peripheral blood lack the function of antigen presentation because of low or negative expression of MHC class II and co‐stimulatory molecules,^67^, ^68^ whereas mouse basophils exhibit antigen presentation and contribute to an increase in Th2 cells. Mouse basophils can acquire the expression MHC class II complexes by trogocytosis, which is the transfer of a part of the cellular membrane including surface proteins via cell‐contact from dendritic cells after treatment of mice with MC903 plus antigen^69^ (Figure 2).

Basophils are also involved in oral allergen‐induced anaphylactic reactions in mice sensitised with TSLP.^70^ Basophils promote differentiation of Th2 cells through production of IL‐4 during *Trichinella spiraris* (Ts), *Heligmosomoides polygyrus* (Hp) and *Litomosomoides sigmodontis* filaria infections.^71^, ^72^, ^73^ In addition, TSLPR‐deficiency but not that of IL‐3R impairs basophils in the draining lymph nodes during Ts infection, leading to reduction of Th2 cells. These suggest that basophil‐derived IL‐4 can enhance Th2 responses in TSLP sensitised mice. In addition, basophils produce IL‐4 to enhance subsequent production of ILC2 in response to TSLP in skin inflammation.^74^ However, bone marrow chimera experiments exhibiting TSLPR‐deficiency on basophils did not confer protection from the inflammation in skin or reduce level of IgE in serum in topical MC903‐induced skin inflammation.^5^ This experiment was conducted by transferring bone marrow cells from *Mcpt8Cre* mice,^2^ which lack basophils by Cre toxicity only in basophils, into either wild type (WT) mice as a control or TSLPRα^−/−^ mice as basophil‐specific TSLPR‐deficient mice. Furthermore, TSLPR‐floxed mice crossed with *Mcpt8* ^*cre*^,^75^ *Mcpt8* ^*cre*^ *Tslpr* ^*fl/fl*^ mice, in a pulmonary inflammation model did not alter the magnitude of the airway inflammation, increase in Th2 cells, or IgE titres in serum after administration of intranasal antigen with topical MC903 treatment in the skin. This suggests that TSLPR on DC and CD4^+^T cells, but not basophils nor ILC2s, are predominantly reactive to TSLP in this type 2 inflammation.^76^ Although the above two independent mouse lines expressing Cre recombinase under gene locus of mouse mast cell protease 8 (mMCP8) have been established,^2^, ^75^ expression of mMCP8 is not critically restricted to basophils; the expression of mMCP8 has been detected in various cell lineages, including a type of macrophage and inflammatory mast cells.^77^, ^78^, ^79^, ^80^ Thus, further studies are needed to clarify the role of basophils and TSLP via methods using mouse lines expressing Cre recombinase in basophils by other genes, such as Cpa3‐Cre mouse^81^ or using antibodies for depletion of basophils such as anti‐FcεRiα antibody (MAR‐1) and anti‐CD200R3 Ab (Ba103 and Ba160, which are the same hybridomas as both of them have the same characters including CDR3 sequences and were generated in the single experiment).^1^, ^2^, ^79^, ^80^, ^82^, ^83^

It is unclear whether human basophils can respond to TSLP. The TSLP risk allele for EoE is associated with an increase in frequency of circulating basophils.^84^ A different single nucleotide polymorphism of the TSLP allele is also associated with a number of basophils.^85^ Two independent groups showed that activated basophils express TSLPR and basophils from patients with allergy upregulate expression of CD203c and release histamine in response to TSLP, which is compatible with IgE‐cross linking and IL‐3.^86^, ^87^ Rhinovirus infection, a risk factor for asthma exacerbations among atopic patients, also increases the expression of TSLPR on basophils.^88^ Furthermore, TSLP promotes the differentiation of human CD34^+^ cells into basophils and eosinophils, which is also one of the ways to enhance basophil‐mediated allergic responses by TSLP. However, Salabert‐Le Guen *et al*.^89^ showed that human basophils from healthy donors and patients with allergies express TSLPR on the cell surface, but do not express of IL‐7Rα and have altered phosphorylation of STAT5. Gambardella *et al*.^15^ detected production of IL‐4, IL‐13 and CXCL8 from human basophils from healthy donors when *in vitro* stimulated with IL‐3 and IL‐33, but not TSLP and IL‐25.

Overall, basophils enhance type 2 immune responses, including allergic responses to TSLP stimulation in both humans and mice, although it remains unclear whether those responses of basophils to TSLP are because of initiation through TSLPR on basophils.

## IL‐33

IL‐33 is an IL‐1 family cytokine identified by bioinformatic analysis of the human genome. The IL‐1 cytokine family contains 11 cytokines including IL‐1α, IL‐1β, IL‐18 and IL‐33.^90^, ^91^ Although IL‐1 cytokines are widely expressed in haematopoietic cells, which are associated with inflammatory responses and host defences,^92^, ^93^ IL‐33 is predominantly produced by epithelial cells such as alveolar type II cells in bronchus and airways, fibroblasts and smooth muscle cells. IL‐33 is hardly detected in mouse blood vessels during homeostasis, whereas human endothelial cells are known to express IL‐33 constitutively and to be a major source of IL‐33 mRNA in inflamed tissues from patients with rheumatoid arthritis, psoriasis and Crohn's disease.^94^, ^95^, ^96^, ^97^ In human, bronchial epithelial cells and endothelial cells are the major sources of IL‐33 in human lungs, whereas IL‐33 is mainly expressed in alveolar type 2 cells in mice.^98^, ^99^, ^100^ During mouse lung development, IL‐33, mainly expressed in alveolar type 2 cells, guides the maturation and immunomodulatory functions of alveolar macrophages through activation of basophils.^100^ The IL‐33 receptor consists of IL‐1 receptor accessory protein (IL‐1RAP) and suppression of tumorigenicity 2 (ST2). IL‐1RAP is a ubiquitous protein, also associated with IL‐1 receptor type 1 (IL‐1R1) and the IL‐36 receptor.^101^ Expression of ST2 is restricted to a subset of T cells, including pathogenic memory Th2 cells (Tpath2), ILC2s, basophils, mast cells, eosinophils and macrophages in humans and mice.^102^, ^103^, ^104^, ^105^, ^106^, ^107^, ^108^, ^109^, ^110^ Although residential Tregs also highly express ST2 compared to circulating Tregs, IL‐33/ST2 axis is dispensable for accumulation and residence in nonlymphoid organs.^111^, ^112^ The IL‐33/ST2‐IL‐1RAP axis induces myeloid differentiation primary response gene 88 (MyD88), IL‐1 receptor‐associated kinase 1 and 4 and TNF receptor associated factor 6.^113^, ^114^ The IL‐33 signalling can inhibit proliferation and induce apoptosis in MIA PaCa‐2, a human pancreatic cancer cell line.^115^

IL‐33 displays a role in homeostatic and inflammatory responses. Adventitial stromal cells in mouse lung parenchyma also express IL‐33 and TSLP, supporting accumulation and activation of ILC2 cells, which may contribute to subsequent inflammatory reactions.^116^ In the bleomycin‐induced lung fibrosis mouse model, airway epithelial cells and alveolar macrophages produce IL‐33 to exacerbate lung fibrosis.^117^ IL‐33 signalling contributes to protection against helminth infections in mice such as *Heligmosomoides polygylus*, *L. sigmodontis*, *Nippostrongylus brasiliensis*, *Strongyloides ratti* and *Trichinella spiralis*.^108^, ^118^, ^119^, ^120^, ^121^, ^122^, ^123^

Elevation of the expression of IL‐33 has been shown in a large number of allergic disorders, including asthma, chronic rhinosinusitis, allergic rhinitis, atopic dermatitis and eosinophilic esophagitis.^124^, ^125^, ^126^, ^127^, ^128^, ^129^ Insulation of cell or tissues, exposure to allergens and infection with nematodes or viruses trigger IL‐33 release in humans and mice. Airborne allergens of the products from fungi including *Alternaria alternata* and *Aspergillus fumigatus*, German cockroach and house dust mite contain proteinase activity. Protease allergens can activate proteinase‐activated receptor‐2 (PAR‐2) through oxidative stress and ATP secretion, resulting in IL‐33 release.^107^, ^130^, ^131^, ^132^ Laundry detergents and surfactants also have activity to increase oxidative stress that induce expression of IL‐33 from airway epithelial cells.^133^ In addition, cigarette smoke induces expression of IL‐33. In the case of COPD, tobacco smoke plus viral damage elevates severity of COPD via increase in IL‐33.^134^ IL‐33 expression in epithelial cells and endothelial cells is also increased by other various stimuli including diesel exhaust particles (DEPs), chitin, silica crystals, hydroxypropyl‐b‐cyclodextrin, viral infection and Toll‐like receptor ligands.^135^, ^136^, ^137^, ^138^, ^139^, ^140^, ^141^, ^142^ Genetic studies have shown significant associations between *IL1RL1* and *IL33* genetic variants and allergic diseases such as asthma, atopic dermatitis and EoE in humans.^143^, ^144^, ^145^, ^146^, ^147^, ^148^, ^149^, ^150^, ^151^ Anti‐IL‐33 antibodies have been tested as therapies for pulmonary diseases including asthma and COPD as a Phase II trial (Table 3). Treatment with anti‐IL‐33 receptor antibodies (CNTO 7160) blocks IL‐33 signalling in mild asthma; however, clinical benefits were not found for patients in this clinical trial.^152^ In the other clinical trial, patients with uncontrolled asthma may have clinical benefits from treatment with anti‐IL‐33 receptor antibodies compared to placebo (GSK37772847), but more studies are needed.^153^ Treatment with Etokimab, an IL‐33 antibody, desensitised peanut‐allergic individuals to efficiently tolerate peanut protein with fewer adverse events and reducing Th2 cytokine production and serum IgE for peanuts compared to placebo in Phase 2a of a clinical trial.^154^ Furthermore, in a Phase 2a study for patients with moderate‐to‐severe atopic dermatitis, administration of Etokimab improved eczema area and severity index (EASI) 50 and EASI75 scores and reduced eosinophils in peripheral blood.^155^

**Table 3:** Clinical trials of biologics targeting IL‐33/ST2 (from ClinicalTrials.gov, accessed 15 November 2024)

Study title	Study status	Conditions	Biologics	Character of biologics	Phases	Identifier
A preliminary study to evaluate PF‐07264660 in healthy participants	Completed	Healthy	PF‐07264660	Tri‐specific monoclonal antibody targeting IL‐4 IL‐13 and IL‐33	Phase 1	NCT05496738
A study to evaluate the safety and tolerability, pharmacokinetics (PK) and pharmacodynamics (PD) of Melrilimab (GSK3772847) in healthy participants	Completed	Asthma healthy volunteers	Melrilimab/GSK3772847	Anti‐IL‐33 receptor mAb	Phase 1	NCT04366349
Study of REGN3500 and Dupilumab in patients with asthma	Completed	Asthma, allergic	REGN3500 Dupilumab	Anti‐IL‐33 mAb anti‐IL‐4 and ‐13 mAb	Phase 1	NCT03112577
A proof‐of‐concept study to assess the efficacy, safety and tolerability of Itepekimab (anti‐IL‐33 mAb) in participants with chronic rhinosinusitis without nasal polyps	Not yet recruiting	Chronic rhinosinusitis without nasal polyps	Itepekimab/SAR440340	Anti‐IL‐33 mAb	Phase 2	NCT06691113
A proof‐of‐concept study to assess the efficacy, safety and tolerability of Itepekimab (anti‐IL‐33 mAb) in participants with non‐cystic fibrosis bronchiectasis	Recruiting	Bronchiectasis	Itepekimab/SAR440340	Anti‐IL‐33 mAb	Phase 2	NCT06280391
Mechanistic study of the effect of Itepekimab on airway inflammation in patients with COPD	Recruiting	COPD	Itepekimab/SAR440340	Anti‐IL‐33 mAb	Phase 2	NCT05326412
A phase II, randomised, double‐blind, placebo‐controlled study to assess MEDI3506 in participants with COPD and chronic bronchitis	Completed	COPD Chronic bronchitis	MEDI3506	Anti‐IL‐33 mAb	Phase 2	NCT04631016
Study to assess the efficacy and safety of MEDI3506 in adults with uncontrolled moderate‐to‐severe asthma	Completed	Asthma	MEDI3506	Anti‐IL‐33 mAb	Phase 2	NCT04570657
Anti‐ST2 (MSTT1041A) in COPD (COPD‐ST2OP)	Completed	COPD exacerbation	MSTT1041A	Anti‐ST2 mAb	Phase 2	NCT03615040
Proof‐of‐concept study to assess the efficacy, safety and tolerability of SAR440340 (anti‐IL‐33 mAb) in patients with moderate‐to‐severe chronic obstructive pulmonary disease (COPD)	Completed	COPD	SAR440340	Anti‐IL‐33 mAb	Phase 2	NCT03546907
Efficacy and safety study of GSK3772847 in subjects with moderately severe asthma	Completed	Asthma	GSK3772847	Anti‐IL‐33 receptor mAb	Phase 2	NCT03207243
A study to learn about two study medicines (PF‐07275315 And PF‐07264660) in people who have moderate to severe atopic dermatitis	Recruiting	Atopic dermatitis	PF‐07264660	Tri‐specific monoclonal antibody targeting IL‐4 IL‐13 and IL‐33	Phase 2	NCT05995964
Efficacy and safety of Tozorakimab in patients hospitalised for viral lung infection requiring supplemental oxygen	Recruiting	Viral lung infection and acute respiratory failure	Tozorakimab/MEDI3506	Anti‐IL‐33 mAb	Phase 3	NCT05624450
Study to assess the efficacy, safety, and tolerability of SAR440340/REGN3500/Itepekimab in chronic obstructive pulmonary disease (COPD)	Active not recruiting	COPD	Itepekimab/SAR440340	Anti‐IL‐33 mAb	Phase 3	NCT04751487
Study to assess the efficacy, safety, and tolerability of SAR440340/REGN3500/Itepekimab in chronic obstructive pulmonary disease (COPD)	Active not recruiting	COPD	Itepekimab/SAR440340	Anti‐IL‐33 mAb	Phase 3	NCT04701983

## IL‐33 and basophils

Basophils and mast cells, which are both granulocytes, are associated with allergic disease in mice and humans expressing ST2, a component of the IL‐33 receptor.^156^ Treatment with IL‐33 activates basophils, leading to enhancement of the degranulation and production of pro‐inflammatory cytokines such as IL‐4, and IL‐13 in mouse.^157^, ^158^ IL‐33 itself also activates human basophils to degranulate and secrete various cytokines such as IL‐1b, IL‐4, IL‐5, IL‐6, IL‐8, IL‐13 and granulocyte‐macrophage stimulating factor.^158^, ^159^, ^160^ IL‐33 activates NF‐kB and p38 MAP‐kinase on human basophils.^159^ Intriguingly, treatment with long‐acting muscarinic antagonist alters the IL‐33‐induced IL‐4 production from basophils in both humans and mice, leading to amelioration of eosinophilic inflammation.^161^ It has been shown that the expression of IL‐33 is elevated in allergic inflammation such as in atopic dermatitis and gastrointestinal inflammation.^148^, ^162^, ^163^ In IL‐33 transgenic mice, basophils activate ILC2 cells to induce inflammatory processes such as atopic dermatitis.^164^ IL‐33 induces activation of mouse basophils through their own ST2 receptor in epicutaneous sensitisation‐induced experimental eosinophilic esophagitis.^165^ Humanised mouse models show that allergen plus IgE‐induced activation of human basophils and can confer allergic gut inflammation through production of platelet‐activating factor (PAF) and histamine.^166^

**Figure 3:**
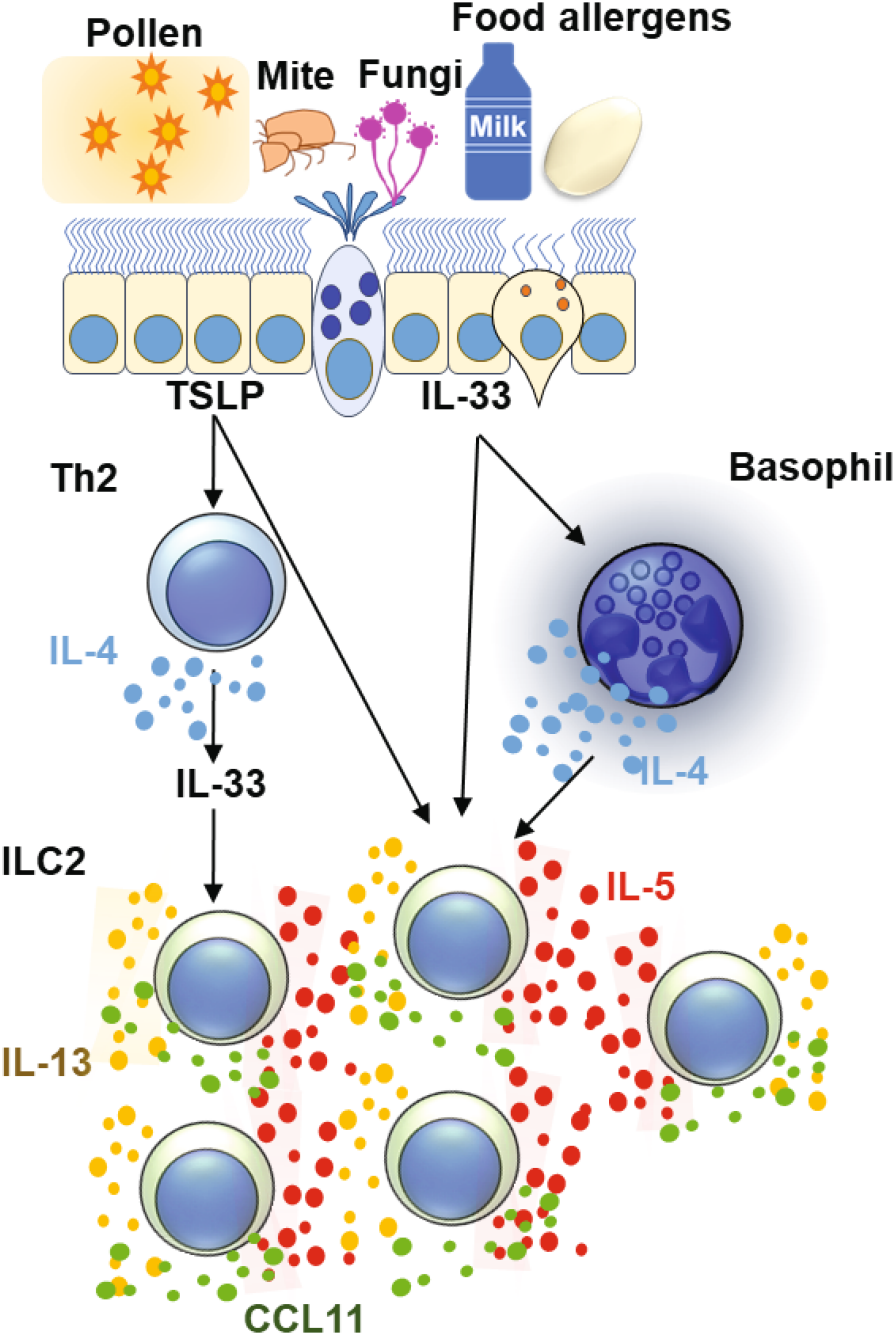
A model of type 2 airway inflammation. Various stimulations induce secretion of type 2 epithelial cytokines from epithelial cells in lung, subsequent activation of basophils and Th2 cells induce expansion and activation of ILC2 cells through production of IL‐4.

In a mouse model of protease‐induced pulmonary inflammation, ILC2 and Th2 cells play a critical role in development phases of eosinophilic inflammation in response to type 2 epithelial cytokines; TSLP is important for Ovalbmin (OVA) immunisation‐induced Th2 cell responses, whereas IL‐33 activates ILC2 cells to promote Th2 cell responses.^167^ The similarities between ILC2 and Th2 cells have been studied in chromatin levels.^168^ In this case, IL‐4 from Th2 cells increase IL‐33‐induced ILC2 responses (Figure 3). In a similar context, basophils produce IL‐4 in response to IL‐33 to increase the production of type 2 cytokines, such as IL‐5 and IL‐13 and the chemokine CCL11 from ILC2s, resulting in elevation of eosinophilic airway inflammation.^169^, ^170^ It has been demonstrated that phosphodiesterase (PDE) 4 is involved in IL‐3 and IL‐33‐induced phosphorylation of ERK and subsequent IL‐4 production from mouse basophils in oxazolone‐induced skin inflammation.^171^ In addition, treatment with long‐acting muscarinic antagonist alters this IL‐33‐induced IL‐4 production from basophils in both humans and mice, leading to amelioration of eosinophilic inflammation.^161^ On the contrary, mast cells suppress pulmonary inflammation induced by treatment with papain through production of IL‐2 to promote Tregs in an IL‐33‐dependent manner.^107^ These results suggest that the role of basophils is distinct from mast cells in IL‐33‐contributed airway inflammation. Collectively, since basophils have unique pathological roles in IL‐33‐contributed allergic diseases, it would be worth targeting both basophils and mast cells in potential therapies for IL‐33‐contributed allergic diseases.

## Future research directions

**Figure 4:**
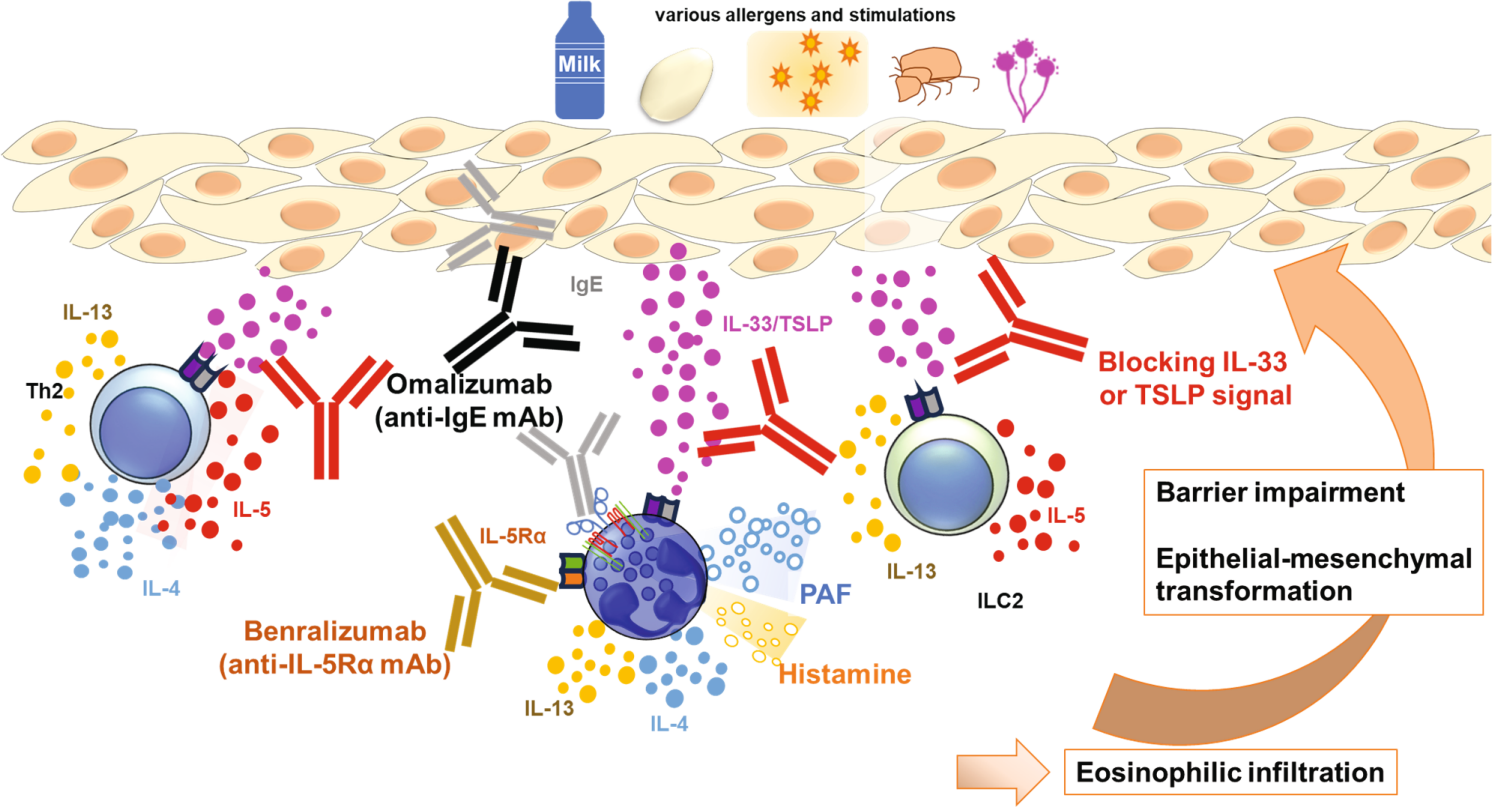
A proposed model of combination therapy with anti‐IL‐33/ST2 mAb or anti‐TSLP mAb plus blocking basophils for gastrointestinal inflammation. Because both type 2 epithelial cytokines and IgE complexes stimulate basophils to exacerbate gut inflammation and Th2 immune responses, it might be beneficial to block both type 2 epithelial cytokines and basophils.^84^, ^165^, ^166^

The studies summarised in this review indicate that type 2 epithelial cytokines of TSLP and IL‐33 play a role as positive modulators in activation of basophils both directly and also indirectly, leading to exacerbation of allergic reactions. In addition, anti‐IgE antibody therapy (Omalizumab) has preventive effects in food allergy (NCT03881696).^172^ Results suggest that synergistic effects may be possible if we block both basophils and type 2 epithelial cytokines in allergic diseases. For example, a combination of antibodies for type 2 epithelial cytokines using anti‐IgE mAb (Omalizumab) or anti‐IL‐5Rα mAb (Benralizumab) treatment, which reduces circulating basophils in EoE^173^ (Figure 4). A tri‐specific monoclonal antibody targeting IL‐4, IL‐13 and IL‐33 (PF‐07264660) and a bifunctional nanobody blocking TSLP and IL‐13 (SAR443765) are in this context. Administration of anti‐IL‐3 Rα (CD123) mAb (Talacotuzumab) also reduces the frequency of basophils in the blood^174^ and reports suggest that the level of production of IL‐3 from phytohemagglutinin‐stimulated peripheral blood mononuclear cells is correlated with improvement of lung function in pre‐school children with asthma.^175^ These future studies will provide novel therapeutic approaches to help patients suffering from allergic diseases.

## Conclusion

We have summarised the role of basophils and type 2 epithelial cytokines, specifically TSLP and IL‐33, in allergic diseases and helminth infections in humans and mice. We hope this manuscript will help clinicians and scientists studying translational and basic science.

## Conflict of interest

The authors declare no conflict of interest.

## Author contributions

**Kazushige Obata‐Ninomiya:** Conceptualization; data curation; formal analysis; funding acquisition; investigation; supervision; validation; visualization; writing – original draft; writing – review and editing. **Tharmalingam Jayaraman:** Visualization. **Steven F Ziegler:** reading and advice.
